# Cecal microbiota of Tibetan Chickens from five geographic regions were determined by 16S rRNA sequencing

**DOI:** 10.1002/mbo3.367

**Published:** 2016-05-02

**Authors:** Xueyan Zhou, Xiaosong Jiang, Chaowu Yang, Bingcun Ma, Changwei Lei, Changwen Xu, Anyun Zhang, Xin Yang, Qi Xiong, Peng Zhang, Shuai Men, Rong Xiang, Hongning Wang

**Affiliations:** ^1^ Animal Disease Prevention and Food Safety Key Laboratory of Sichuan ProvinceKey Laboratory of Bio‐resources and Eco‐environmentMinistry of Education“985 Project” Science Innovative Platform for Resource and Environment Protection of Southwestern ChinaCollege of Life SciencesSichuan UniversityChengduChina; ^2^ Institute of Poultry ScienceAcademy of Sichuan Animal Husbandry ResearchChengduChina

**Keywords:** Cecal microbiota, Daheng broiler chickens, DGGE, high‐throughput sequencing, Lohmann egg‐laying hens, Tibetan Chicken

## Abstract

Tibetan Chickens should have unique gastrointestinal microbiota because of their particular habitats. Thus, the aim of this study was to investigate the cecal microbiota of Tibetan Chickens from five typical high‐altitude regions of China. Lohmann egg‐laying hens (LMs) and Daheng broiler chickens (DHs) were chosen as controls. The cecal bacterial populations of Tibetan Chickens were surveyed by high‐throughput sequencing (HTS) of the bacterial 16S rRNA hypervariable region V3‐V4 (16S rRNAV3‐V4) combined with community‐fingerprinting analysis of the 16S rRNA gene based on polymerase chain reaction‐denaturing gradient gel electrophoresis (PCR‐DGGE). The results revealed that the majority of cecal microbiota differed between the Tibetan Chicken and LM/DH. The microbial communities in the cecum were composed of 16 phyla, 28 classes, 36 orders, 57 families, 101 genera, and 189 species. Represented phyla were *Bacteroidetes* (>47%), *Firmicutes* (>18.8%), *Spirochaetae* (>0.3%), and *Proteobacteria* (>0.4%). *Bacteroides* and the *RC9* gut group were the two most abundant genera. There were relatively more *Christensenellaceae*,* Subdoligranulum*,* Spirochaeta*, and *Treponema* in Tibetan Chickens, whereas there were more *Phascolarctobacterium*,* Faecalibacterium*,* Megamonas*, and *Desulfovibrio* in LMs and DHs. The cecal microbiota of Tibetan Chicken have slightly diverged due to exposure to different geographic environments. Differences in the intestinal bacterial communities of Tibetan Chicken and LM/DH were noted.

## Introduction

The Tibetan Chicken is a high‐altitude breed inhabiting the Qinghai‐Tibetan Plateau at 2200–4100 meters and adjacent regions, including Tibet and Qinghai, Sichuan, and Yunnan of China. The breed originated in Tibet and immigrated to other places. Their rearing history is approximately 1000 years. The Tibetan Chicken lived cage free in a high altitude, hypoxic environment, which resulted in its unique adaptation to the plateau, characterized by resistance to hypoxia and roughage. We speculated that the Tibetan Chicken was geographically distributed on the plateau at different altitudes, allowing the establishment of unique intestinal microflora, which would significantly contribute to their survival in the region. Studies, mostly in chickens, have found that intestinal microbiota are dynamic and complex and play an important role in nutrition (Mead 1989; Apajalahti et al. 2004), immunity (Brisbin et al. 2008; Yilmaz et al. 2014), and detoxification (Young et al. 2007) in avian hosts and have an enormous impact on the maintenance of health (Stanley et al. 2014). The Tibetan Chicken is a native chicken breed used for both eggs and meat. Lohmann egg‐laying hen (LM), an egg layer, was introduced into China in 1989. Daheng broiler chicken (DH) is a local broiler breed of Sichuan. Over 300 days, we collected old healthy female Tibetan Chicken adults in five high‐altitude areas (Lhasa in Tibet, Ganzi and Aba in Sichuan, Haibeimenyuan in Qinghai, and Diqing in Yunnan) to study their intestinal microflora. LM and DH were used as controls (Lu et al. 2003; Scupham et al. 2008). Because the parent birds were difficult to collect, all samples were derived from commercial chickens. This study was designed to investigate the unique structure of the intestinal flora in Tibetan Chickens under their normal habitat conditions. If Tibetan Chickens had been bred for a long time by scale farming in the plains, it is possible that their unique microflora would be altered and reestablished as an adaptive microbial community structure, but some species may be lost forever.

## Materials and Methods

### Animals and sample collection

The collected chickens had no history of gut infectious disease or antimicrobial administration in the preceding 3 months and were fed an antibiotic‐free diet. They were healthy female Tibetan Chicken adults over 300 days old from Lhasa, Ganzi, Aba, Qinghai, and Diqing in China. Compared with other portions of the bowel, the effect of diet on cecal microbiota is minimal. Cecal microbiota have been compared with intestinal microflora (Barnes 1972). A total of 15 individuals (*n *=* *15) from each site were sampled, and one cecum was aseptically removed from each chicken. The cecum was wrapped in foil and immersed in liquid nitrogen. Subsequently it was transported cold (in dry ice) to the laboratory. Samples were stored at −80°C until processed. Samples from Lohmann laying hens and DHs were collected in the same manner.

### DNA extraction and DNA qualification

The cecal contents of five chickens were pooled to reduce interindividual variation. A prior study indicated that the optimal sample size for evaluating the composition of the cecal microbiota of chickens was five birds per pool (Zhou et al. 2007). Total bacterial DNA from stool samples was extracted using a QIAamp DNA stool kit (Qiagen, Hilden, Germany) according to the manufacturer's protocol. The DNA was suspended in distilled, deionized water (ddH_2_O). DNA quantity and quality were measured on a NanoDrop ND‐1000 spectrophotometer (Thermo Fisher Scientific, Waltham, Massachusetts, EUA). DNA integrity after extraction was determined using 0.8% agarose gels in (Tris/Acetic acid/EDTA) buffer. After detection, equimolar concentrations of three parallel community DNA samples from one site were pooled to reduce possible heterogeneity and to obtain data that were representative of an ‘average’ sample from a site (Lamendella et al. 2011).

### PCR amplification of 16S ribosomal RNAV3 and DGGE

The V3 region of 16S ribosomal RNA was amplified. The primers 341F (5′‐ATTACCGCGGCTGCTGG‐3′) and 534R with GC clamps (5′‐CGCCCGCCGCGCGCGGCGGGCGGGGCGGGGGCACGGGGGGCCTA CGGGAGGCAG CAG‐3′) against the V3 region of 16S rRNA genes (positions 339–539 in the *E. coli* gene) were used (Yin et al. 2010). For denaturing gradient gel electrophoresis (DGGE), the p534 reverse primer was modified at the 5′ end with a 40‐bp GC‐rich clamp sequence that terminated the gel migration of the products in a urea/formamide concentration gradient (Holben et al. 2004). The touchdown PCR program was performed in a S1000 thermocycler (Bio‐Rad, Hercules, CA) according to a previous procedure (Li et al. 2007). The reaction mixture consisted of 2 *μ*L of template DNA, 12.5 *μ*L of iProof 2× master mix (containing buffer, nucleotides, and iProof enzyme), and 1.5 *μ*L of each primer (final concentration of 0.5 mmol/L), for a final volume of 25 *μ*L with the addition of ddH_2_O. Amplification of the products was performed in an Eppendorf Mastercycler using the following conditions: an initial denaturation for 5 min at 95°C, followed by 10 cycles of denaturation for 30 sec at 95°C, primer annealing for 30 sec from 60 to 55°C (at each temperature for two cycles), and primer extension for 30 sec at 72°C. Another round of 20 denaturation cycles for 30 sec at 94°C, annealing for 30 sec at 50°C, and elongation for 30 sec at 72°C was followed by a final elongation step (72°C) of 5 min. All amplicons (2 *μ*L) were analyzed on GreenView‐stained 1× TAE agarose gels before being used for DGGE.

DGGE analysis of the 16S rRNA gene amplicons was conducted as previously described (Muyzer et al. 1993). Briefly, DGGE was performed using a Bio‐Rad DCodeTM Mutation Detection System (Bio‐Rad, Hercules, CA) according to the manufacturer's instructions. Amplicons were separated in a 40–60% gradient for 12 h at 80 V in Tris/Acetic acid/EDTA (TAE) buffer at a constant temperature of 60°C. Gels were photographed using a Molecular Imager^®^ Gel DocTM XR system (Bio‐Rad). The similarity of the PCR‐DGGE profiles was analyzed using Quantity One 4.6.9 software, with a match tolerance of 2% (Version 4.6.9; BIO‐RAD, Hercules, CA). Principal Coordinate analysis (PCoA) was performed on the band‐matching matrix.

### PCR amplification of 16S rRNAV3‐V4 and high‐throughput sequencing

Broad‐range PCR amplification of the V3–V4 hypervariable region of the 16S rRNA gene was performed using a 338F forward‐primer formulation‐targeting domain bacteria, including *Planctomycetales* and *Verrucomicrobiales*, along with an 806R reverse primer with 8‐bp barcodes to facilitate multiplexing (Caporaso et al. 2012). The primer pairs used were 338F 5′‐ACTCCTACGGGAGGCAGCAG‐3′ and 806R 5′‐GGACTACHVGGGTWTCTAAT‐3′. PCR reactions (20 *μ*L) were performed using 4 *μ*L of 5× FastPfu Buffer, 2 *μ*L of 2.5 mmol/L dNTPs, 0.8 *μ*L of forward primer (5 *μ*mol/L), 0.8 *μ*L of reverse primer (5 *μ*mol/L), 0.4 *μ*L of FastPfu Polymerase, 10 ng of template DNA, for a final volume of 20 *μ*L with the addition of ddH_2_O. The reactions were run on an ABI GeneAmp^®^ 9700 (Applied Biosystems, Foster City, CA, USA). The PCR cycling conditions included three steps: a. 1 × (3 min at 95°C); b. 27 × (30 sec at 95°C, 30 sec at 55°C, and 45 sec at 72°C); and c. 10 min at 72°C and 10°C until terminated manually. The efficiency of PCR amplification for each sample was verified by agarose gel electrophoresis. The amplified, individually barcoded, 16S rRNA amplicons from each sample were sequenced on the Illumina Miseq PE300 platform, which provided millions of reads up to 300 × 2 bp in length.

Information analysis of the sequence data was accomplished via four programs. First, the sequencing data were preprocessed. Sequences were assigned to samples according to specific barcodes and primers. The output sequence file was statistically analyzed using publically available software packages and databases. Trimmomatic (v0.33: http://www.usadellab.org/cms/?page=trimmomatic) was used for trimming. FLASH (Fast Length Adjustment of SHort reads, Version 1.2.11: http://ccb.jhu.edu/software/FLASH/), a read pre‐processing tool, merged the paired‐end reads from fragments and generated >10 bp overlapped reads from Illumina pair‐end reads. Raw reads must be quality filtered to some degree before downstream analysis. Filtering typically involved removing some reads based on length, quality score, ambiguous bases, homopolymers, and chimeric sequences. Through quality control using Qiime (Version 1.7.0, http://qiime.org/scripts/split_libraries_fastq.html), high‐quality data (clean reads) were acquired. Clean reads were BLASTed in the Gold database (http://drive5.com/uchime/uchime_download.html). UCHIME Algorithm (http://www.drive5.com/usearch/manual/uchime_algo.html) detected chimeric sequences, removed the chimera, and obtained valid data (effective reads). Second, operational taxonomic units (OTUs) were identified as particular bacterial taxa. All effective reads from each sample were initially clustered into OTUs by Uparse software (Uparse v7.0.1001, http://drive5.com/uparse/) at 97% sequence identity. A single sequence of the highest appearing frequency of the OTUs was selected as the representative sequence of OTUs, which was BLASTed in the primary databases, including RDP Classifier (Version 2.2, http://sourceforge.net/projects/rdp-classifier/), GreenGenes (http://greengenes.lbl.gov/cgi-bin/nph-index.cgi), and the SILVA database (http://www.arb-silva.de). Third, in alpha‐diversity analyses, the Chao1 estimator, ACE estimator, Shannon index, Simpson index, and Good's coverage were calculated using Mothur (version v.1.30.1 http://www.mothur.org/wiki/Schloss_SOP#Alpha_diversity). Relative abundance was determined with R software (Version 2.15.3). Fourth, in beta‐diversity analysis, unifrac distances were calculated by the Bray–Curtis of Qiime software, and a hierarchical clustering tree of samples was constructed by UPGMA (Unweighted Pair‐group Method with Arithmetic Means). Heatmap were performed using vegan, vegdist, and hclust by R software.

## Results

### DGGE analysis of the 16S rRNAV3 region

**Figure 1:**
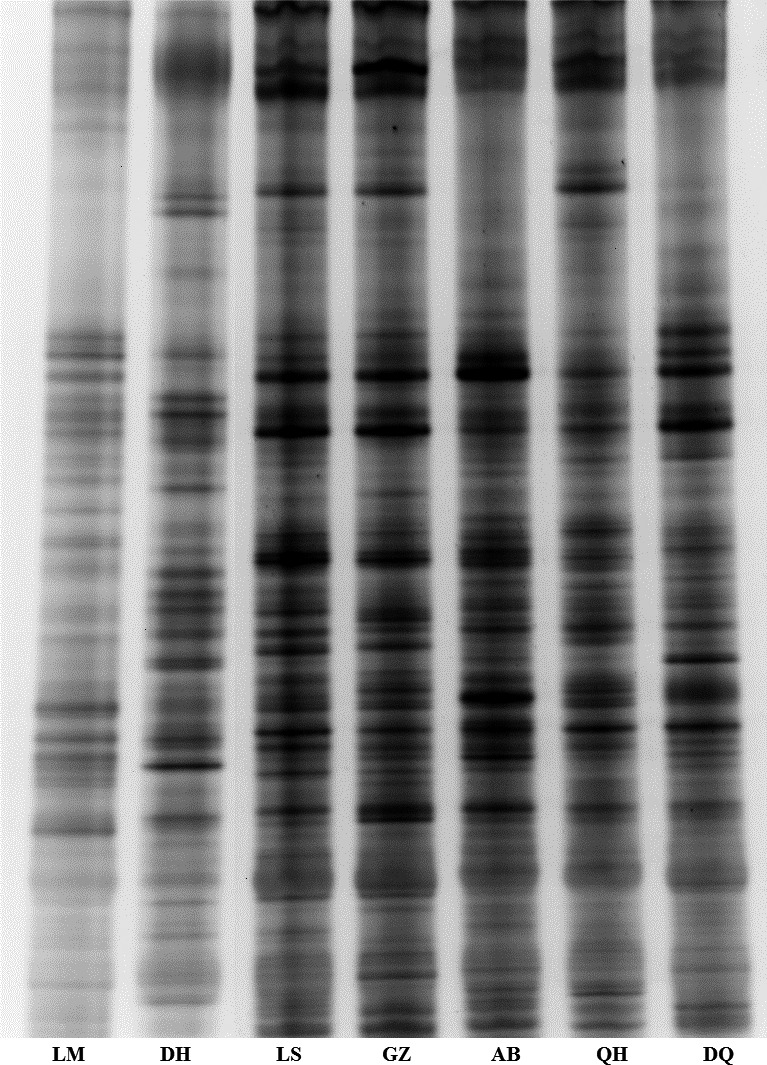
Denaturing gradient gel electrophoresis band profiles of the V3 region of 16S rRNA produced from the cecal bacterial communities of seven types of chicken. LM, Lohmann laying hens; DH, Daheng broiler chickens; LS, Lhasa Tibetan Chicken; GZ, Ganzi Tibetan Chicken; AB, Aba Tibetan Chicken; QH, Qinghai Tibetan Chicken; and DQ, Diqing Tibetan Chicken.

The 16S rRNAV3 genes from the cecal bacteria of collected chickens were amplified via PCR. DGGE analyses for the V3 region were performed and exhibited a diverse banding pattern (Fig. 1) indicative of a rich bacterial community; several shared bands were observed in the samples. DGGE band profiles revealed the existence of intergroup variations among the cecal bacterial flora of the Tibetan Chickens (LS, GZ, AB, QH, and DQ) collected from the five geographic sites and the Lohmann laying hens and DHs. These significant variations were presumed to result from variations in the original regions. Closer geographic distances demonstrated more similar DGGE fingerprints.

### Principal coordinate analysis (PCoA)

**Figure 2:**
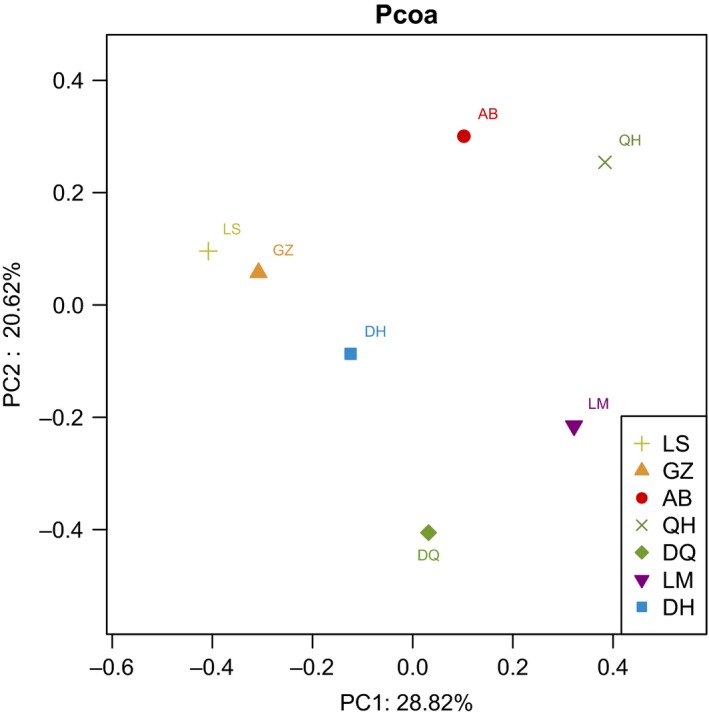
Principal coordinate analysis of seven samples based on denaturing gradient gel electrophoresis fingerprinting. Points of different colors or shapes represent a group of samples under different environmental conditions. The horizontal and vertical axis scale indicates relative distances. PC1 and PC2 represent the potential factors that caused a microbial composition shift of the samples.

PCoA was used to study the similarities or differences in the community composition of the samples. Quantity one 4.6.9 software was used to digitize the brightness of the DGGE band profile of each lane into numerical values. Then, the various values were analyzed by Canoco for Windows 4.5 software. The results showed that points of seven samples were distributed in different coordinates within a plane. Three geographic clusters could be recognized via PCoA analysis based on DGGE fingerprinting (Fig. 2). Lhasa Tibetan Chicken (LS) and Ganzi Tibetan Chicken (GZ) showed high similarity with DH; Aba Tibetan Chicken (AB) approached Qinghai Tibetan Chicken (QH); and Diqing Tibetan Chicken (DQ) approached LM. The distance showed the degree of similarity in the bacterial communities among the seven subjects.

### Rarefaction analysis of 16S rRNAV3‐V4 metagenomic sequences

**Figure 3:**
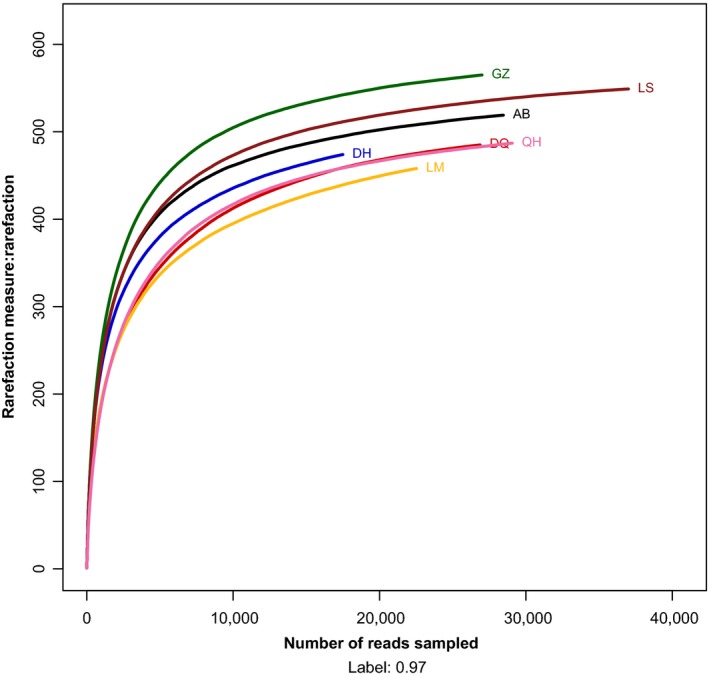
Rarefaction curve of the seven samples. Horizontal axis: the amount of effective sequencing data; vertical axis: the observed number of operational taxonomic units

Rarefaction is a technique for assessing species richness from sampling results that allows the calculation of species richness for a given number of individual samples based on the construction of so‐called rarefaction curves (Amato et al. 2013). On the left, a steep slope indicates that a large fraction of the species diversity remains to be discovered. If the curve becomes flatter to the right, a reasonable number of individual samples have been taken, and more intensive sampling is likely to yield only few additional species (Gotelli and Colwell 2001). Thus, using Mothur software to perform rarefaction analysis for OTUs (97% similarity), we detected the sequencing depth of the sample. The calculated rarefaction curves for the seven samples leveled off from the 1:1 interval and indicated that the amount of sequencing data for the samples was reasonable (Fig. 3).

### Alpha‐diversity analysis indices

When studying microbial diversity in community ecology, alpha‐diversity analysis can reflect community richness and diversity of a sample, and beta‐diversity (between‐habitat diversity) can reflect differences in microbial community structure between samples. The composition of the PCR products in terms of OTUs and taxonomical groups was used for estimating the associated alpha diversity and beta diversity of the analyzed samples. Calculated alpha‐diversity indices included the Chao1/ACE richness estimator, the Shannon and the inverse Simpson diversity indices, and the sequencing depth index–Good's coverage estimator, which was calculated for assessing the percentage of diversity captured by the devoted sequencing effort. The Shannon diversity index (H) calculated at 97% sequence identity varied between H = 3.94 and H = 4.95, with the lowest diversity found in the QH sample and the highest found in the GZ sample (Table 1).

**Table 1:** Alpha‐diversity indices of the bacterial communities

Sample ID	Average length (bp) of sequences	Effective reads	0.97 Similarity					
			OTU	Ace estimator	Chao1 estimator	Shannon index	Simpson index	Good's coverage
LS	439.88	37007	549	570	578	4.79	0.0255	0.998865
GZ	436.96	27001	565	586	588	4.95	0.0210	0.998370
AB	438.32	28462	519	539	539	4.82	0.0286	0.998595
QH	441.42	29068	487	512	527	3.94	0.0826	0.998245
DQ	441.51	26875	485	504	504	4.40	0.0328	0.998214
LM	441.34	22533	458	504	504	4.48	0.0265	0.996849
DH	440.10	17499	474	514	525	4.91	0.0170	0.996286

### OTU distribution from a Venn plot

**Figure 4:**
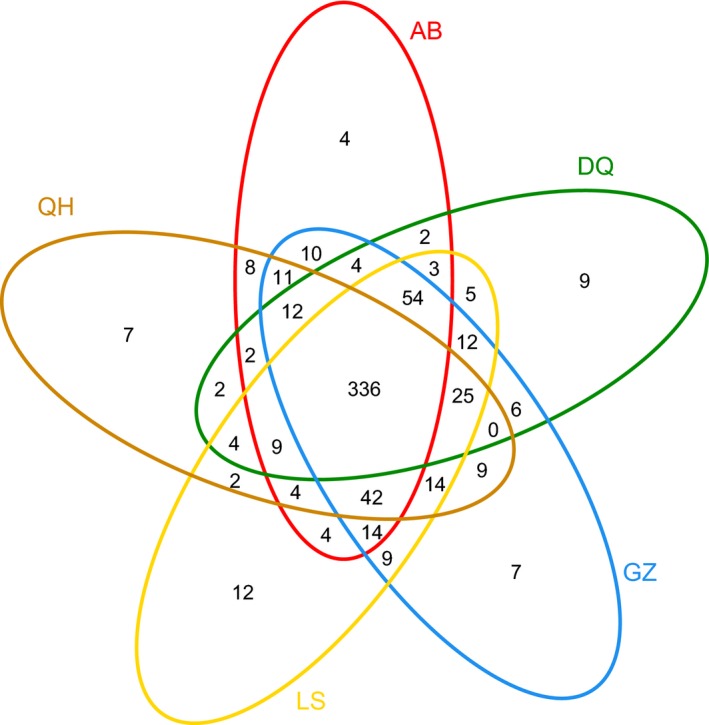
Venn plot of operational taxonomic unit (OTU) distribution. Each ellipse represents one of the samples. The overlapping regions between the ellipses represent shared OTUs between samples. The value for each region represents the number of OTUs corresponding to the region.

Sequences of amplicons of the bacterial 16S rRNAV3‐V4 region obtained by high‐throughput sequencing (HTS) were also analyzed by an OTU‐based approach after clustering together with at least 97% sequence identity. To determine richness and diversity, OTUs were identified at genetic distances of 3% (species level), 5% (genus level), and 20% (phylum level) by using quality sequences with a read length of 300–600 nucleotides per sample. A Venn plot was used to show shared and unique OTUs found in each plotted group. This procedure more intuitively shows the similarity and overlap of the OTU composition of the samples (Fouts et al. 2012). The cecal microbiota of Tibetan Chickens diverged slightly, and a greater variety of overlaps (336 OTUs) (core) were shared by all of the plotted groups. Unique OTUs (12, 7, 9, 4, and 7) were found among LS, GZ, DQ, AB, and QH, respectively (Fig. 4).

### Structure and diversity of bacterial community profiles

**Figure 5:**
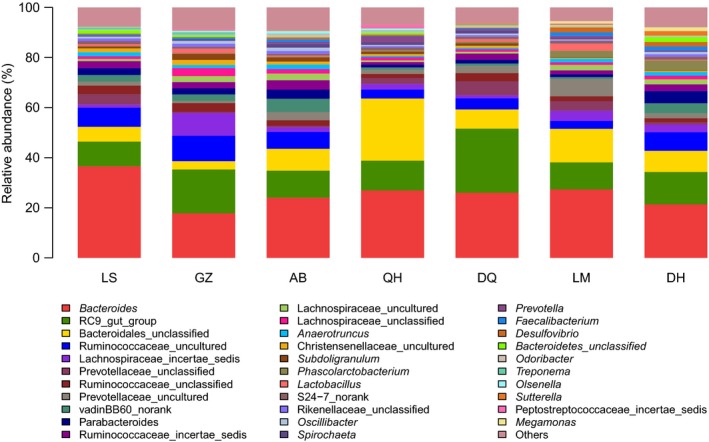
Relative abundance of the dominant bacteria at the genus level. Lhasa Tibetan Chicken (LS), Ganzi Tibetan Chicken (GZ), Aba Tibetan Chicken (AB), Qinghai Tibetan Chicken (QH), and Diqing Tibetan Chicken (DQ) indicate LS, GZ, AB, QH, and DQ Tibetan Chickens, respectively; Lohmann egg‐laying hen and Daheng broiler chicken indicate laying hens and broiler chickens, respectively.

All samples showed good diversity, with a mean coverage of 0.9979 for the samples at a genetic distance of 0.03. The composition of the microbial communities included 101 different bacterial genera and 189 species (33 genera are shown in Fig. 5) by taxonomic classification of the obtained 16S rRNA gene sequences. At the genus level, some differences were detected in the abundance of the bacterial communities among the various ceca (Fig. 4). Most notably, *Bacteroidetes* (>17%) and the *RC9* gut group (>9.8%) were the most abundant groups among all samples. *Christensenellaceae* were relatively more abundant in the Tibetan Chickens, as were *Subdoligranulum*,* Spirochaeta*, and *Treponema*. However, *Phascolarctobacterium*,* Faecalibacterium*,* Desulfovibrio*, and *Megamonas* were more abundant in LM and DH (Table 2).

**Table 2:** Abundance of genera in the bacterial communities

Genus	Abundance (%)						
	LS	GZ	AB	QH	DQ	LM	DH
*Christensenellaceae*	1.53	2.07	1.18	0.87	0.82	0.34	0.45
*Subdoligranulum*	0.75	2.35	1.65	1.01	0.94	0.23	0.21
*Spirochaeta*	0.38	0.65	1.50	1.04	1.20	0.23	0.23
*Treponema*	0.87	1.06	0.21	0.26	0.25	0	0.14
*Phascolarctobacterium*	0.23	0.36	0.36	0.49	0.71	2.62	4.01
*Faecalibacterium*	0.39	0.55	1.11	0.23	0.40	1.51	1.57
*Megamonas*	0	0	0	0	0.02	0.88	1.50
*Desulfovibrio*	0.36	0.17	0.74	0.44	0.21	1.98	1.79

### Heatmap of bacterial community

**Figure 6:**
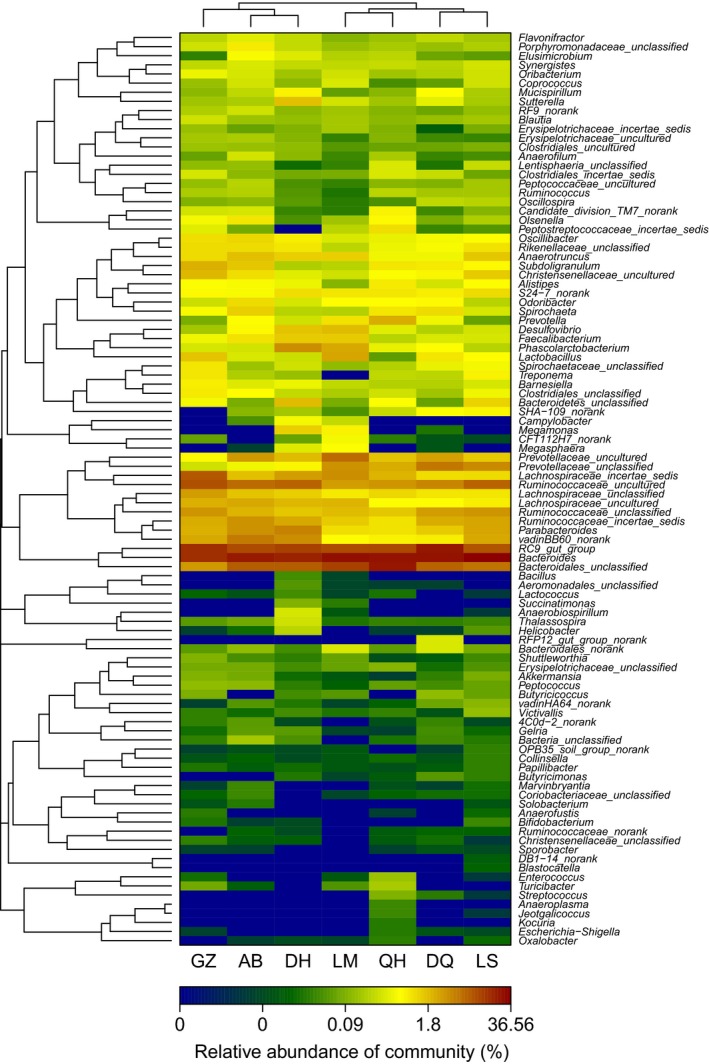
Heatmap of the distribution of the 100 most abundant bacterial genera. Hierarchical Ward‐linkage clustering based on Spearman correlation coefficients of the genus proportions. Genera were filtered for a subject prevalence of at least 30% within a sample. Subjects and genera (100) were visualized and clustered by the vertical tree. A colored block represents the genus abundance of a sample.

The heatmap can reflect the actual similarities and differences in community composition of the samples. The abundance of the top 100 genera shared by all of the samples is displayed (Fig. 6). The branch structure could be used to describe and visualize the similarities and differences in the relationships among multiple samples (Jiang et al. 2013). The OTU‐level data had the highest resolution for differentiating bacterial communities in the samples (Srinivasan et al. 2012). First, the distance between samples was calculated by the linkage algorithm described in community composition and structure, that is, hierarchical clustering analysis based on a beta‐diversity distance matrix. Then, a clustering tree was constructed by a UPGMA algorithm. GZ, AB, and DH were similar in composition at the genus level. QH and LM, and LS and DQ were in the two branches.

### Nucleotide sequence accession numbers

The nucleotide sequences obtained were deposited in the Sequence Read Archive database of NCBI with the accession numbers SRR2756648, SRR2764842, SRR2764855, SRR2764070, SRR2764489, SRR2755827, and SRR2529360.

## Discussion

To better understand the taxonomy of the bacterial communities in the Tibetan Chicken cecum, we conducted HTS of the 16S rRNAV3‐V4 region with semiquantitative PCR‐DGGE. A previous study suggested that a combination of two or more methods may be required to reliably ascertain the microbial constituents of a microbial consortium (Kashinskaya et al. 2015). PCR‐DGGE has long been used for analyzing relative community structure (Muyzer et al. 1993). However, this process is subject to various inherent errors and PCR bias (Neilson et al. 2013), such as inaccurate abundance estimation or poor data reproducibility (Bent et al. 2007). It is semiquantitative (Zoetendal et al. 2004) and can only detect taxa of >1% abundance (Muyzer 1999). HTS has an unprecedented potential to detect rare species that usually cannot be detected with classical molecular approaches, such as PCR‐DGGE. In this study, the community differences among the samples were first observed by PCR‐DGGE. Then, we used the HTS method to analyze the composition of the microbial community and diversity of the samples at each taxonomic level. Our results confirmed the presence of the principal bacterial species in the cecum of chickens as assessed by PCR‐DGGE, but with greater resolution, and provided a quantitative assessment specific for the genus‐level composition that was not possible by simple analyses of gel‐banding patterns.

This study further supported a previous work published in Cell. Lean individuals had higher levels of the highly heritable taxon *Christensenellaceae* (Goodrich et al. 2014). We investigated the genus *Christensenellaceae*, which had two‐ to fourfold or higher abundance in the cecal microbiome of Tibetan Chickens than in LM/DH. The experimental Tibetan Chickens were small in size and lean, with a low percentage of abdominal fat (PAF). The dressed weight (DW) was 963.57 ± 12 g, and the PAF was 0.25 ± 0.0533%. LM and DH were large and obese. The DWs of LM and DH were 1608.35 ± 152 g and 2018.67 ± 254.55 g, and the PAFs were 5.2 ± 1.2% and 3 ± 1.14%, respectively. *Christensenella* (within the phylum *Firmicutes*, class *Clostridia*, and order *Clostridiales*) could be isolated from human feces (Morotomi et al. 2012). This taxon was saccharolytic and negative for catalase, oxidase, and urease; hydrolysis of aesculin and gelatin; nitrate reduction; and indole production. The end products of glucose fermentation were acetic acid and a small amount of butyric acid. It should be noted that Tibetan Chickens live in high‐altitude regions and that LM/DH are low‐altitude chickens. Whether *Christensenella* contributed to the plateau adaptability of the Tibetan Chickens is a subject for further study, and *Christensenella* in the chicken gut should be investigated.

Different intestinal microbiota can impact host health and metabolism. The abundance of *Subdoligranulum*,* Spirochaeta*, and *Treponema* was higher in the Tibetan Chickens than in LM and DH, possibly due to the early‐stage feeding of antibiotics to LM and DH, although antibiotic feeding was discontinued after that time (Wise and Siragusa 2007). *Treponema* were present at low levels in the cecum after ASP250 (chlortetracycline, sulfamethazine, and penicillin) treatment (Looft et al. 2014). *Subdoligranulum* was a novel genus from human feces in the *Clostridium leptum* group bacteria and was strictly anaerobic, Gram negative, and coccoid. Glucose and some other carbohydrates could be fermented (Holmstrøm et al. 2004). A rich diversity of free‐living *spirochaeta* was found in Indian habitats (Shivani et al. 2015). *Spirochaetes* sp. strain JC202 was isolated from the gut of a termite (Isoptera) (Sravanthi et al. 2015). The strain of *spirochaeta* present in Tibetan Chickens should be identified. *Treponemas* are spoilage bacteria and are chemoheterotrophic, free living, and anaerobic. Most are pathogens that cause skin diseases (Klitgaard et al. 2014). *Phascolarctobacterium, Faecalibacterium, Desulfovibrio*, and Megamonas were less abundant in the Tibetan Chicken than in LM and DH. *Phascolarctobacterium* is an asaccharolytic, succinate‐utilizing bacterium (Watanabe et al. 2012). *Faecalibacterium* is an anti‐inflammatory bacterium. Virginiamycin led to significant enrichment of the genus *Faecalibacterium* in the cecum of mature broilers (Neumann and Garret 2015), but its richness was reduced in patients with inflammatory bowel disease (Lopez‐Siles et al. 2015). *Megamonas* (order, *Clostridiales*; family, *Acidaminococcaceae*) helped to ferment various carbohydrates in the gut (Chevrot et al. 2008). *Desulfovibrio*, class *Delta‐proteobacteria*, are a species of sulfate‐reducing bacteria that metabolize the sulfate moiety of sulfated mucins. An altered abundance of *Desulfovibrio* has been reported in ulcerative colitis (UC). There was a weak but significant negative correlation between the abundance of sulfated mucins and the *Desulfovibrio* burden. The abundance of *Desulfovibrio* was increased by a significant decrease in sulfomucin in UC (Lennon et al. 2014). Therefore, backyard‐bred Tibetan Chickens had some opportunistic pathogens, whereas mucosal inflammation risks existed in the intestinal tracts of LM and DH bred on large‐scale farm in cages.

**Figure 7:**
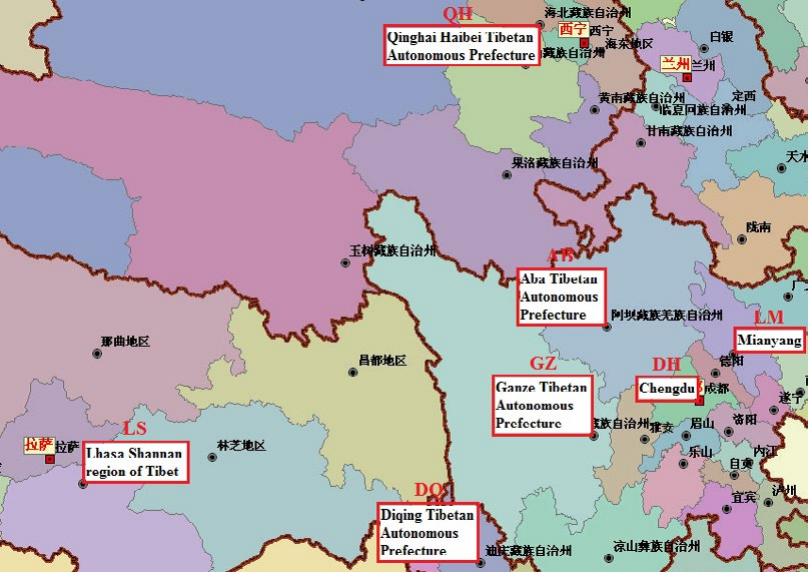
Map of the sampling sites. Samples and sampling sites are marked. Geographic locations surrounded by a red box were the sampling sites.

Cluster analysis of the cecal microbes indicated that the differences in the composition of the intestinal microbiota between the Tibetan Chickens and the LM/DH chickens were associated with geographic conditions. In the Tibetan Chickens, the degree of similarity of the intestinal microbiota was correlated with geographic distance (Fig. 7). Tibetan Chickens are reared in backyards. Their feed may not be exactly the same, but the effect of feed on the microbial composition of the cecum should be minimal (Zhu et al. 2002). Because of long‐term geographic dispersion, separation occurred in the intestinal microbial composition of the Tibetan Chicken. Clustering at the genus level more reliably indicated the actual composition of the samples than at the phylum level. The genus‐level heatmap showed that GZ and AB were clustered because both lived in Sichuan province and thus were nearest in distance. It was more convenient to immigrate from LS (Lhasa) to DQ than from GZ (Ganzi). DQ was relatively closer to LS. Thus, LS and DQ were clustered. QH, in northwest China, was the farthest from the other four places and was isolated. However, the composition of QH was similar to that of LM. After many years of immigrant life in Sichuan, the cecal microbes of GZ and AB were similar to those of DH. Similar studies have been conducted in other birds. Adult passerine birds showed differences in their cloacal microbial communities due to geographic location, diet, and season (Klomp et al. 2008). In Adelie penguins (Pygoscelisadelie), fecal flora similarity was negatively correlated with both host genetic distance and geographic distance (Banks et al. 2009). Thus, the microbiota composition of an individual was altered depending on where they lived within a single biogeographic region, in a homogeneous cohort where any other confounding factors have limited effects.

## Conflict of interest

No conflict of interest is declared.
